# Investigating the mechanism of chloroplast singlet oxygen signaling in the *Arabidopsis thaliana accelerated cell death 2* mutant

**DOI:** 10.1080/15592324.2024.2347783

**Published:** 2024-05-03

**Authors:** Matthew D. Lemke, Alexa N. Abate, Jesse D. Woodson

**Affiliations:** The School of Plant Sciences, University of Arizona, Tucson, AZ, USA

**Keywords:** Chloroplast, photosynthesis, programmed cell death, reactive oxygen species, signaling, singlet oxygen

## Abstract

As sessile organisms, plants have evolved complex signaling mechanisms to sense stress and acclimate. This includes the use of reactive oxygen species (ROS) generated during dysfunctional photosynthesis to initiate signaling. One such ROS, singlet oxygen (^1^O_2_), can trigger retrograde signaling, chloroplast degradation, and programmed cell death. However, the signaling mechanisms are largely unknown. Several proteins (e.g. PUB4, OXI1, EX1) are proposed to play signaling roles across three *Arabidopsis thaliana* mutants that conditionally accumulate chloroplast ^1^O_2_ (*fluorescent in blue light* (*flu*), *chlorina 1* (*ch1*), and *plastid ferrochelatase 2* (*fc2*)). We previously demonstrated that these mutants reveal at least two chloroplast ^1^O_2_ signaling pathways (represented by *flu* and *fc2*/*ch1*). Here, we test if the ^1^O_2_-accumulating lesion mimic mutant, *accelerated cell death 2* (*acd2*), also utilizes these pathways. The *pub4–6* allele delayed lesion formation in *acd2* and restored photosynthetic efficiency and biomass. Conversely, an *oxi1* mutation had no measurable effect on these phenotypes. *acd2* mutants were not sensitive to excess light (EL) stress, yet *pub4–6* and *oxi1* both conferred EL tolerance within the *acd2* background, suggesting that EL-induced ^1^O_2_ signaling pathways are independent from spontaneous lesion formation. Thus, ^1^O_2_ signaling in *acd2* may represent a third (partially overlapping) pathway to control cellular degradation.

## Introduction

To thrive while rooted to the ground, plants have acquired the ability to sense changes in their environment and acclimate. While plants employ diverse mechanisms to achieve such plastic physiologies, it is clear that they can use their chloroplasts (photosynthetic plastid organelles) to sense and respond to multiple types of abiotic and biotic stresses. This is due, in part, to chloroplasts being the site of photosynthesis. Such high energy metabolism is prone to producing reactive oxygen species (ROS), particularly under stress conditions such as drought, excess light (EL), salinity, and pathogen attack,^1–3^ While these ROS can be toxic and lead to the damage of macromolecules in the chloroplast, they also act as signaling molecules.^4^ One ROS in particular, singlet oxygen (^1^O_2_), is predominantly associated with chloroplasts (unlike superoxide and hydrogen peroxide (H_2_O_2_), which are made in multiple cellular compartments) and produced primarily at photosystem II during photosynthesis due to excited triplet-state chlorophylls interacting with molecular oxygen,^5-7 1^O_2_ is known to trigger changes in nuclear gene expression (e.g., retrograde signaling),^8,9^ selective chloroplast degradation (i.e., chloroplast quality control),^10,11^ and programmed cell death (PCD).^12,13^ Due to the extremely short half-life of ^1^O_2_ (<1 μsec,^14^ the bulk of this ROS is expected to remain within the chloroplasts in which it is made, necessitating the existence of signaling cascades. However, how ^1^O_2_ triggers such signaling and leads to these effects predominantly remains an open question in plant cell biology.

To study these signals, researchers have primarily relied on three *Arabidopsis thaliana* mutants that conditionally accumulate ^1^O_2_ in chloroplasts. The first characterized, *fluorescent in blue light* (*flu*),^15^ accumulates the tetrapyrrole chlorophyll precursor protochlorophyllide (Pchlide) in the dark. Upon a shift to light, this photosensitizing molecule leads to a rapid burst of ^1^O_2_ within chloroplasts that initiates a signal from the grana margins,^16^ which leads to retrograde signaling and PCD.^9,13^ A second mutant *chlorina* (*ch1*), cannot produce chlorophyll *b*, leaving the PSII reaction center without a protective antenna and sensitive to EL stress.^12^ Under EL, *ch1* mutants produce a large amount of ^1^O_2_ at PSII within the grana core leading to retrograde signaling and PCD.^12,17^ This ^1^O_2_ can also lead to EL acclimation and increase EL tolerance, a process that requires METHYLENE BLUE SENSITIVITY 1 (MBS1) in Arabidopsis^18^ and green algae.^19^ Finally, a third mutant, *plastid ferrochelatase 2* (*fc2*), accumulates the chloroplast tetrapyrrole intermediate protoporphyrin IX (Proto) immediately after dawn.^10^ Like Pchlide, free Proto also leads to the generation of ^1^O_2_ in the light, although the exact location of where the ^1^O_2_ is generated is unknown.

In all three mutants, the accumulation of chloroplast ^1^O_2_ leads to the induction of similar sets of nuclear marker genes, rapid bleaching of photosynthetic tissue, and eventual cell death.^9,10,12^ In the case of *fc2*, these signals also lead to selective^11^ and wholesale chloroplast degradation,^10^ possibly depending on the bulk level of ^1^O_2_. Remarkably, these effects are not due to the toxicity of ^1^O_2_ per se, but to a genetically programmed response to its accumulation. In all three mutants, genetic suppressors have been identified through forward and reverse genetic approaches and many of these suppressor mutations can block signaling without reducing ^1^O_2_ levels. ^1^O_2_ signaling in *flu* can be blocked by mutations in *EXECUTOR1* (*EX1*)^13^ and *EX2* ,^20^ which encode two chloroplast thylakoid-localized proteins. Signaling in *flu* is also blocked by mutations in *CRYTOCHROME1* (*CRY1*),^21^ which encodes a nuclear-/cytoplasmic-localized blue light photoreceptor. In *ch1*, mutations affecting *OXIDATIVE INDUCIBLE SIGNAL1* (*OXI1*) block ^1^O_2_ signaling.^17^ OXI1 is a nuclear-localized Ser/Thr kinase, originally identified for its role in pathogen defense.^22^ Finally, several mutations that block ^1^O_2_ signaling in *fc2* have been identified.^10,23,24^ One mutation, *pub4–6*, affects the E3 ubiquitin ligase Plant U-Box 4 (PUB4), which may be involved (directly or indirectly) with the ubiquitination of proteins associated with the chloroplast envelope during photo-oxidative stress.^25^ Such ubiquitination may be a mechanism by which photo-damaged chloroplasts are targeted for degradation.^26^

At first glance, these three mutants appear to use the same ^1^O_2_ signaling pathway. They all induce PCD and regulate the expression of the same photo-oxidative stress nuclear marker genes. However, it is not known if these mutants represent one or multiple chloroplast signaling pathways. To this end, we recently used a meta-analysis of previously published whole transcriptome data sets of the three mutants to determine if they regulate the same set of genes^27^ due to generating the same ^1^O_2_ signal. We observed that they share a small core transcriptional response to ^1^O_2_ stress (36 genes), but maintain unique patterns. This result opened the possibility that these mutants use different^1^O_2_ signals that report on specific stresses. Next, we combined the suppressor mutations described above with ^1^O_2_-producing backgrounds in which they were not originally isolated.^27^ We then tested the ability of these suppressor mutations to block or alter ^1^O_2_ signaling in these new genetic backgrounds. Our results suggested that these mutants may represent two distinct ^1^O_2_ signaling pathways: one represented by *flu* (involving EX1/EX2 and CRY1), and one represented by *fc2* and *ch1* (involving PUB4 and OXI1). This was based on the observation that mutations that block signaling in *flu* (*ex1*/*ex2* and *cry1*) did not directly block signaling in *fc2*. The *cry1* mutation also did not block signaling in *oxi1*, while *ex1*/*ex2* were previously shown not to affect ^1^O_2_ signaling in this mutant.^17^ In addition, genetic suppressors of *fc2* (*pub4–6*) and *ch1* (*oxi1*) did not affect ^1^O_2_ signaling in *flu*. At the same time, *fc2* and *ch1* seemed to share the same pathway. The *pub4–6* mutation was able to block EL-induced PCD in *ch1*, and the *oxi1* mutation was able to block PCD in *fc2* adult plants (but not seedlings). Together, these results pointed to chloroplasts using at least two separate ^1^O_2_ pathways to induce PCD and retrograde signaling.

A fourth, less characterized mutant, *accelerated death 2* (*acd2*), has also been shown to accumulate ^1^O_2_, leading to spontaneous lesion formation in adult leaves.^28,29^ The ACD2 protein is needed to convert red chlorophyll catabolite (RCC) to primary fluorescent chlorophyll catabolite (pFCC) during chlorophyll catabolism.^30^ In *acd2* mutants, the photosensitive tetrapyrrole RCC accumulates, which is likely responsible for the burst of ^1^O_2_.^31^ The site of ^1^O_2_ production in *acd2*, however, is not clear. ACD2 is expected to be active near PSII,^32^ but also localizes to mitochondria under stress conditions.^33^ The latter observation may explain why ^1^O_2_ has been shown to accumulate in *acd2* mitochondria.^34^ Nonetheless, overexpression of *ACD2* has been shown to delay the hypersensitive response (HR) induced by *Pseudomonas syringae* infection, suggesting the regulation of tetrapyrrole catabolism may play a role in a pathogen defense response.^33^ In citrus, an ACD2 homolog is a target of the *Candidatus* Liberibacter Asiaticus effector protein SDE15, and this interaction suppresses the plant’s hypersensitive response.^35^ Consequently, ACD2-related ^1^O_2_ production may act as a mechanism by which plants can trigger PCD in response to pathogen attack.

Previously, it was shown that *acd2* mutants do not produce a *flu*-like signal, as *ex1*/*ex2* and *cry1* mutations were unable to block spontaneous cell death phenotypes in *acd2*.^33^ To test if *fc2* may share a pathway with *acd2*, we previously generated an *acd2 pub4–6* double mutant and showed that it had delayed lesion formation,^27^ suggesting that the ^1^O_2_ produced in *acd2* mutants triggers PCD through a PUB4-related mechanism. However, it is unknown if this is the same ^1^O_2_ pathway also shared with the *ch1* mutant. Here, we follow-up on these studies by testing the role OXI1 plays during lesion formation in the *acd2* mutant. We show that OXI1 is dispensable for spontaneous lesion formation in *acd2*, but not for EL-induced ^1^O_2_-dependent) lesion formation, suggesting these responses may represent two separate signaling pathways to induce PCD.

## Methods

### Plant material and growth conditions

The wild type (wt) used in this study was *Arabidopsis thaliana* ecotype *Columbia* (Col-0). T-DNA line GABI_355H08 (*oxi1–1*)^36^ from the GABI-Kat collection,^37^ *pub4–6* ,^10^ and *acd2–2* ^29^ were described previously. The line expressing plastid localized YFP (*35S::TobaccoRBCS*(1–79)-YFP) was described previously.^38^ Additional information on these lines is listed in Table S1. Double mutants were created by crossing single mutants. Genotypes were confirmed by extracting DNA using a CTAB-based protocol^39^ and using PCR-based markers to detect the presence of a T-DNA (*oxi1*) or a SNP (*acd2* and *pub4–6*) as previously described.^27^ Primer sequences are listed in Table S2.

Seedlings were germinated on half-strength Linsmaier and Skoog medium pH 5.7 (Caisson Laboratories North Logan, UT) in 0.6% micropropagation type-1 agar powder (PlantMedia, CAS:9002-18-0) and grown as previously described.^40^ Seedlings were first grown in constant light conditions in a Percival^Ⓡ^ model CU-36L5 plant tissue culture chamber (with fluorescent bulbs) with a light intensity of ~ 70 photons µmol photons m^−2^ sec^−1^ at 21°C. Seven-day-old seedlings were carefully transferred to soil (PRO-MIX LP15) supplemented with fertilizer (Jack’s Classic All Purpose fertilizer, 6.7 mL of 300 g/L solution per flat), and growth was continued in a LED plant growth chamber (Hettich PRC 1700) at ~ 110–120 µmol photons m^−2^ sec^−1^ at 21°C and 60% relative humidity, set to diurnal cycling light (16 h light/8 h dark) conditions. Photosynthetically active radiation was measured using a LI-250A light meter with a LI-190 R-BNC-2 Quantum Sensor (LiCOR).

### Excess light treatments

21-day-old plants were exposed to EL treatments of 1450–1550 µmol photons m^−2^ sec^−1^ white light at 10°C (to offset for excess heat generated by the EL panel) for 24 h in a Percival LED 41L1 chamber (with SB4X All-White SciBrite LED tiles) as previously described.^41^ The average leaf temperature was measured using an Etekcity Lasergrip 630 Infrared Thermometer and was consistently between 19°C and 20°C.

### Confocal microscopy

Leaf sections were imaged on the adaxial side using a Zeiss 880 inverted confocal microscope. The excitation/emissions wavelengths used for chlorophyll autofluorescence, and yellow fluorescent protein (YFP) fluorescence were 633 nm/638-721 nm and 514 nm/519-620 nm, respectively. YFP and chlorophyll were scanned on separate tracks with truncated emissions spectra to prevent signal bleed-over. Images were processed using ZEN BLUE (Zeiss) software and analyzed in IMAGEJ/FIJI (https://imagej.net).

### Measuring reactive oxygen species

^1^O_2_ was measured with Singlet Oxygen Sensor Green dye (SOSG, Molecular Probes) as previously described.^41^ Briefly, leaf disks (4 mm) were cut (using a cork borer) from true leaves (#’s 5–6 not containing lesions) and placed in 250 µl of 50 mM phosphate buffer, pH 7.5, wrapped in foil, and returned to the growth chamber for 2 days, which has been shown to be sufficient to detect ^1^O_2_ in *acd2*^*31*^. One hour before light exposure (subjective dawn) on day three, SOSG solution (final concentration 1 μg/μl SOSG solution) was added under dim green light, vacuum infiltrated for 30 min, and incubated for another 30 min. Leaf disks were then returned to the growth chamber and light exposure for 2 h and then imaged. SOSG fluorescence was measured with a Zeiss Axiozoom 16 fluorescent stereo microscope equipped with a Hamamatsu Flash 4.0 camera and a GFP fluorescence filter. The average SOSG signal (fluorescence per mm^2^ of each leaf disk was quantified using ImageJ.

H_2_O_2_ was measured using 3,3′-diaminobenzidine tetrahydrochloride (DAB) as described.^42^ Briefly, leaves (# 5–6 not containing lesions) were submerged in DAB solution (1 mg/ml DAB solution + Tween 20 (0.05% v/v)), vacuum infiltrated in the dark, and placed in a tube rotator overnight. The following day, the DAB stain was removed and replaced with a bleaching solution (ethanol:acetic acid:glycerol, 3:1:1). Tubes were then boiled for 15 min, and the bleaching solution was replaced before an overnight incubation. Leaves were imaged and the average DAB signal (fluorescence per mm^2^ of each leaf was quantified using ImageJ.

### Biomass measurements

Mean dry weight biomass was assessed by collecting total aerial tissue of 57-day-old plants (after seed set) in envelopes. Plant tissue was thoroughly dried for 3 days in a 65°C oven before weighing.

### Chlorophyll fluorescence measurements

Chlorophyll fluorescence measurements were conducted with whole plant rosettes as previously described.^40^ Briefly, the maximum quantum yield of PSII (Fv/Fm) measurements was obtained from whole plant rosettes. Plants were dark acclimated in a FluorCam chamber (Closed FluorCam FC 800-C/1010-S, Photon Systems Instruments) for at least 15 min. And measurements were obtained according to the manufacturer’s protocol. Individual leaf measurements were extracted from whole plant images.

### Graphical model creation

Figure 3 is created using online BioRender software (https://biorender.com/).

## Results and discussion

**Figure 1.**
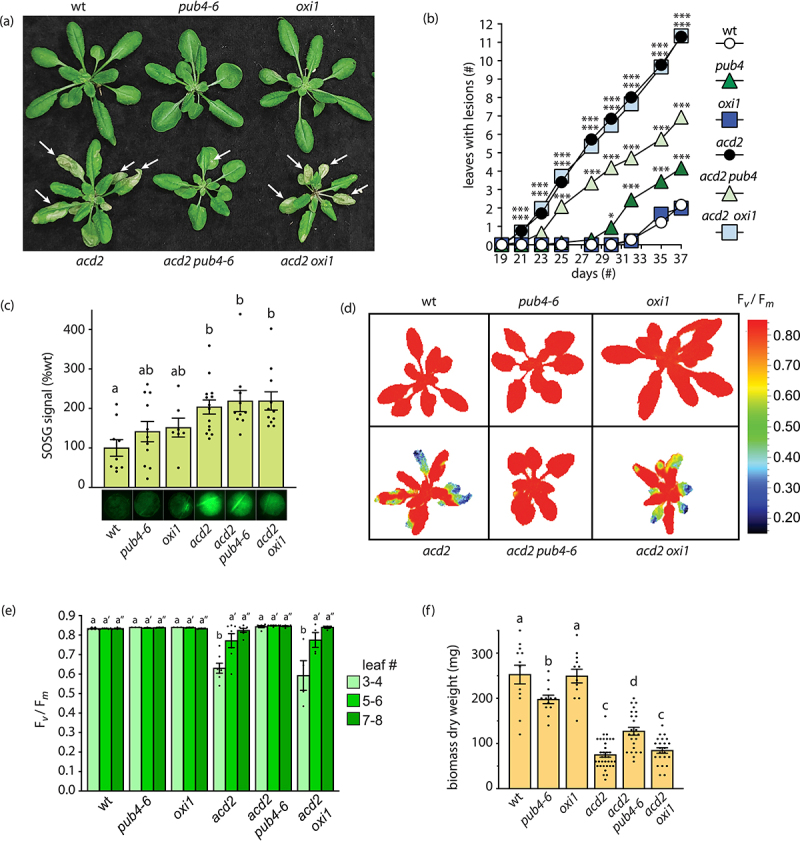
Assessing the effect of *pub4–6* and *oxi1* on lesion formation in *acd2*. (a) Representative images of 24-day-old plants. White arrows indicate lesions. (b) assessment of mean lesion formation (number of rosette leaves with lesions) in plants between 19 and 37 days old (n ≥ 18 plants). (c) singlet oxygen (^1^O_2_) accumulation in leaves (#’s 5–6) from 24-day-old plants. Shown are mean singlet oxygen sensor green (SOSG) intensities per leaf disc (*n* ≥ leaves from individual plants). Below graph are images of representative leaf discs (d) Representative images of 24-day-old plants showing maximum photosynthetic efficiency (F_v_/F_m_) values. (e) mean F_v_/F_m_ values taken from leaves in panel D. Leaves were separated based on age (#’s 3–4, 5–6, or 6–7), measured, and averaged per plant (n ≥ 3 leaf groups from individual plants). F) mean dry weight biomass (mg) from total aerial tissue of 57-day-old plants (n ≥ 12). All plants were grown in cycling light conditions (16 h light/8 h dark) with 125 µmol photons m^−2^ sec^−1^ white light at 21°C. Statistical analyses were performed with a one-way ANOVA. In panel B, a Dunnett’s multiple comparisons posttest was used to test variation between genotypes relative to wt at each time point (* = *P* ≤ .05, ** = *P* ≤ .01, *** = *P* ≤ .001). In panels C, E, and F, a Tukey’s multiple comparisons post-test were used to compare variation between genotypes. Different letters above bars indicate significant differences between genotypes (*P* ≤ .05). In panel E, separate statistical analyses were performed for the different leaf groups, and the significance for groups #5–6 and #7–8 are indicated by single (ʹ) or double (ʹʹ) prime symbols, respectively. Graph bars indicate ±SEM. Closed circles indicate individual data points.

Our previous study indicated that ^1^O_2_ produced in *acd2* mutants triggers spontaneous lesion formation and PCD through a PUB4-related mechanism.^27^ However, it is unknown if this is the same ^1^O_2_ pathway also shared with the *ch1* mutant. To test this, here we generated an *acd2 oxi1* double mutant and assessed lesion formation in adult leaves. As shown in Figure 1(a,b), lesions begin to spontaneously form in *acd2* mutants after 21 days in cycling light (16 h light/8 h dark) conditions with 125 µmol photons m^−2^ sec^−1^ white light at 21°C. This was particularly evident in older leaves (#’s 3 and 4). As expected, the *pub4–6* mutation delayed this lesion formation at least up through 37 days. However, the *oxi1* mutation had no discernable effect on this phenotype, and *acd2 oxi1* mutants exhibited a similar number of lesions to *acd2*. We also measured ^1^O_2_ accumulation in these lines using SOSG (Figure 1(c)). In agreement with previous findings,^31^ *acd2* mutants overaccelerated ^1^O_2_. However, neither *pub4–6* or *oxi1* mutations had any significant effect on this accumulation, suggesting that *pub4–6* was not reducing lesion formation simply by reducing ROS levels.

Next, we tested the effect of the *acd2* mutation on photosynthesis by measuring maximum photosynthetic efficiency (F_v_/F_m_) in leaves. As shown in Figure 1(d,e), *acd2* mutants begin to exhibit lower F_v_/F_m_ values after 24 days in older leaves (#’s 3 and 4), but not newer leaves (#’s 5–6 or 7–8), indicating a degree of photosynthetic stress and loss of chloroplast function. This effect was completely reversed by the *pub4–6* mutation, but not by the *oxi1* mutation. Finally, we tested the final dry weight biomass of these mutants to assess what effect the spontaneous lesions in *acd2* have on the final yield. *acd2* mutants had significantly reduced final biomass (dry weight) after seed set compared to wt, presumably due to having impaired photosynthesis during the reproductive phase (Figure 1(f)). This was partly reversed by the *pub4–6* mutation, but not by the *oxi1* mutation. Notably, the *pub4–6* mutant did not lead to a general increase in biomass, as shown by the reduced biomass (compared to wt) of the single mutant. Together, this shows that the *pub4–6* mutation protects cells and/or chloroplasts to maintain photosynthesis in *acd2* mutants, which positively impacts the final biomass yield. The *oxi1* mutation, on the other hand, had no measurable effect on any of these phenotypes. Thus, spontaneous lesion formation in *acd2* may be distinct from EL-induced ^1^O_2_ signaling, which involves both PUB4 and OXI1 in wt and *ch1* mutants.^12,27^

**Figure 2.**
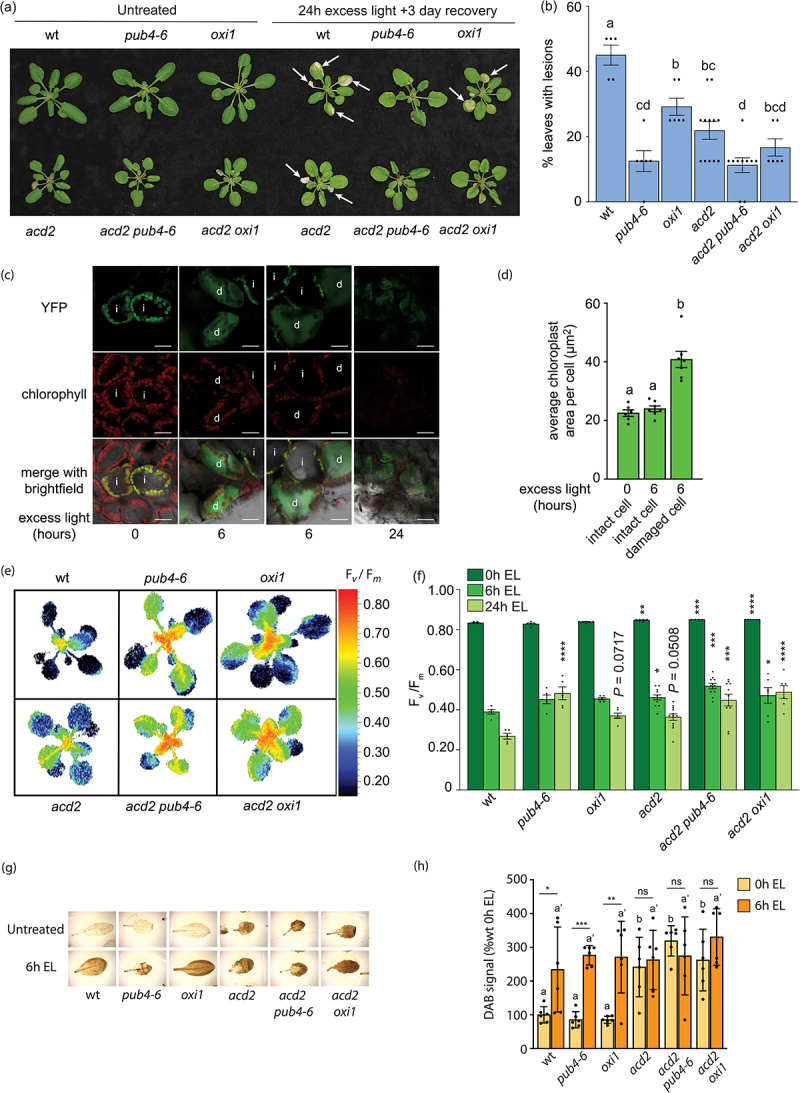
Assessing the tolerance of *acd2* mutants to excess light stress. Plants were grown for 21 days in cycling light (16 h light/8 h dark) conditions at 21°C and then exposed to excess light (EL) at an intensity of 1450–1550 µmol photons m^−2^ sec^−1^ white light at 10°C. (a) Representative images of plants, either unexposed (left) or exposed to EL stress for 24 hours and allowed to recover for three days (right). White arrows indicate lesions. (b) mean % of leaves with lesions (ratio of leaves with observable cell death/healthy leaves) immediately after 24 h EL exposure (*n* ≥ 5 plants). (c) shown are representative laser scanning confocal microscopy images of wt plants expressing plastid localized YFP after 0, 6, or 24 h of EL. Cells with intact (i) and degrading (d) chloroplasts are indicated. Scale bars = 30 μm. (d) mean chloroplast areas from cells with intact or degrading chloroplasts. Areas were estimated based on chlorophyll autofluorescence. Only cells expressing YFP were considered (*n* ≥ 6 cells from 3 plants). (e) Representative images of stressed plants (immediately after 24 h of EL) showing maximum photosynthetic efficiency (F_v_/F_m_) values. (f) mean F_v_/F_m_ values calculated from whole plant rosettes after 0 h, 6 h, or 24 h EL exposure (*n* ≥ 5 plants). (g) shown are representative leaves (#5–6) from plants before and after 6 h EL stress stained with 3,3′-diaminobenzidine tetrahydrochloride (DAB). (h) shown are the mean values of DAB intensity of the leaves in panel G (*n* = 6 leaves from individual plants). Statistical analyses in panels B, D, F, and H were performed with one-way ANOVAs. In panels B, D, and H, Tukey’s multiple comparisons posttest was used to compare variation between genotypes. Different letters above bars indicate significant differences between genotypes (*P* ≤ .05). In panel H, separate analyses were performed for each time point and the significance for 6 h is indicated by prime (ʹ) symbols. Difference between time points for a genotype were performed by student’s t-tests. In panel F, a Dunnett’s multiple comparisons posttest was used to test variation between genotypes within a treatment relative to wt. * = *P* ≤ .05, ** = *P* ≤ .01, *** = *P* ≤ .001, **** = *P* ≤ .0001, ns = *P* ≥ .05. Error bars = ± SEM. Closed circles indicate individual data points.

To test if this is the case, we grew plants for only 21 days (before we observed spontaneous lesion formation in *acd2*) and exposed them to EL (1450–1550 µmol photons m^−2^ sec^−1^ white light) at 10°C for 6 h or 24 h to induce ^1^O_2_ and photo-oxidative stress.^12,17^ No lesions were detected at 6 h, but as shown in Figure 2(a,b), wt plants started to develop lesions by 24 h. To determine if these lesions correlated with chloroplast rupture, we also exposed wt plants expressing plastid-localized YFP to EL. Prior to EL, all YFP co-localized with chlorophyll, indicating the expected chloroplast-localization (Figure 2(c)). After 6 h of EL, the distribution of YFP changed markedly in some cells. In these cases, YFP no longer associated with chloroplasts, and was instead distributed throughout the cell, indicating vacuolar collapse and the onset of PCD. YFP-less chloroplasts were also swollen in size (Figure 2(d)), a hallmark of degrading organelles.^11,43^ After 24, little YFP or chlorophyll was detected, suggesting that chloroplast degradation was complete (Figure 2(c)). Thus, EL stress was able to induce chloroplast degradation reminiscent to^1^O_2_-triggered degradation observed in *fc2* ,^10^ supporting the hypothesis that EL and^1^O_2_ induce overlapping pathways.^44,45^

Next, we tested if our mutants behaved differently in EL. As previously shown,^12,27,41^ the *pub4–6* and *oxi1* mutations delayed EL-induced lesion formation. Unexpectedly, the *acd2* mutants also had delayed lesion formation, but the *acd2 pub4–6* and *acd2 oxi1* double mutants had even fewer lesions (*acd2* vs. *acd2 pub4–6*; *p* = .008, *acd2* vs. *acd2 oxi1*, *p* = 0.244 (student’s t-tests)). Next, we measured the effect of EL on F_v_/F_m_ values in whole rosettes. As shown in Figure 2(e,f), 6 h and 24 h of EL reduced F_v_/F_m_ in wt. As expected,^27^ *pub4–6* retained slightly higher values at 24 h. Surprisingly, before and after EL, *acd2* mutants also had slightly increased F_v_/F_m_ values compared to wt, indicating an increased tolerance to EL. This tolerance was further increased in the *acd2 pub4* and *acd2 oxi1* double mutants and the effect was additive (*acd2* vs. *acd2 pub4*-6, *p* = .016; *acd2* vs. *acd2 oxi1*, *p* = .001 (student’s t-tests)).

To test if any plants were experiencing altered photo-oxidative stress, we measured ROS production. SOSG (the only commercially available in vivo marker specific to ^1^O_2_^46^ is auto-activated under higher light intensities and cannot be used to measure ^1^O_2_ under EL.^47^ As such, we decided to measure H_2_O_2_, which is also produced in chloroplasts under EL stress.^48^ In wt, H_2_O_2_ significantly accumulated after 6 h of EL (Figure 2(g,h)). All other tested mutants had similar levels of H_2_O_2_ after 6 h, suggesting that none of the mutations delayed lesion formation by limiting ROS production. Interestingly, all lines with the *acd2* mutation had significantly higher levels of H_2_O_2_ prior to EL stress, compared to wt, which was not significantly changed by 6 h of EL stress.

Together, these EL experiments indicate that EL-induced lesions in 21-day-old plants may be mechanistically distinct to the spontaneous *acd2* lesions observed after 24 days, the latter involving^1^O_2_ signaling through a unique PUB4-related pathway. Yet, the EL pathway appears to still be active in 21-day-old *acd2* mutants as *pub4–6* and *oxi1* delay EL-induced lesion formation and the drop in Fv/F_m_ values in this mutant. Under EL stress, chloroplasts swell and rupture in a way reminiscent of ^1^O_2_-induced chloroplast degradation in *fc2* mutants (Figure 2(c,d)), which may involve autophagosome-independent microauthophagy.^10,11^ It is unclear if a similar process occurs in *acd2* mutants. Further structural studies of chloroplast degradation under different stresses and in different genotypes will be useful in determining if multiple types of degradation machinery can be involved, or if a single type can be induced by different signals.

Notably, *acd2* was not sensitive to EL stress, at least before the formation of spontaneous lesions. This was surprising as *acd2* potentially accumulates chlorophyll breakdown intermediates near PSII that can produce ^1^O_2_,^32^ particularly under EL stress. However, such ROS, including the constitutively high levels of H_2_O_2_ we observed (Figure 2(g,h)), may lead to a stress-acclimation response leading to reduced EL-sensitivity. Whether such a response involves MBS1,^18^ or altered levels of tetrapyrrole intermediates is unknown.

**Figure 3.**
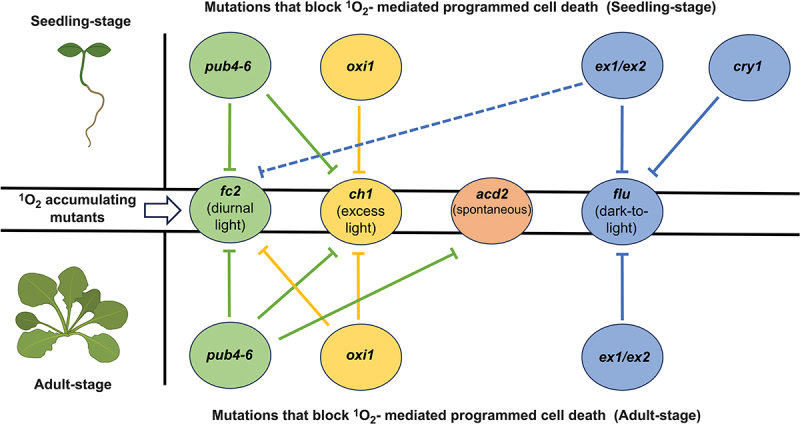
Differential effects on programmed cell death by mutations that block singlet oxygen-signaling. A model summarizing the differential effects on programmed cell death (PCD) by mutations known to block singlet oxygen ^1^O_2_)-signaling in *Arabidopsis thaliana*. Circles in the center row represent four ^1^O_2_ accumulating mutants and conditions in which ^1^O_2_-induced PCD can occur: *plastid ferrochelatase 2* (*fc2*) (green) (diurnal light), *chlorina* (*ch1*) (yellow) (excess light), *accelerated cell death 2* (*acd2*) (orange) (spontaneous), and *fluorescent in blue light* (*flu*) (blue) (dark-to-light). Circles on the top and bottom rows represent secondary mutations (*pub4–6*, *oxi1*, *ex1*/*ex2*, *cry1*) and their ability to block ^1^O_2_ signaling (PCD) in the seedling (top) and adult stages (bottom). Circles sharing the same color as the ^1^O_2_ accumulating mutants indicate the mutant background in which these signaling mutants were first discovered. Solid lines and dashed lines indicate direct and indirect suppression of ^1^O_2_ signaling, respectively (*ex1 ex2* was shown to indirectly block PCD in *fc2* seedlings by reducing ^1^O_2_ levels.^27^

Together, our results further define the ^1^O_2_ signaling pathways in plants and show that PUB4 and OXI1 represent two partially overlapping pathways in addition to the EX1/CRY1-dependent pathway identified in *flu* mutants (Figure 3). While *oxi1* can block ^1^O_2_ signaling that leads to PCD in *fc2* and *ch1*, *pub4–6* can block such signaling in these mutants as well as spontaneous lesions in the *acd2* mutant. As *acd2* may be producing ^1^O_2_ to combat pathogens^35^ or within mitochondria,^34^ it is tempting to conclude that PUB4 may be able to act more broadly in ROS signaling, possibly through immune responses that lead to PCD or hypersensitivity-like responses to pathogens. This is in line with recent reports linking PUB4 to basal defense pathways.^49–51^ and tolerance to heat stress in the dark.^41^ Such work underlines the complexity of chloroplast signaling, which may indicate how flexible these organelles are in sensing their environments and providing information for the cell.

## Supplementary Material

Lemke et al 2024 SOM_revised.docx

## Data Availability

All data generated and analyzed during this study are included in this published article.
