# Supplementary material for: Investigating the mechanism of chloroplast singlet oxygen signaling in the Arabidopsis thaliana accelerated cell death 2 mutant
Source: Plant Signal Behav. 2024 May 3;19(1):2347783. doi: 10.1080/15592324.2024.2347783 (PMC11073415; doi:10.1080/15592324.2024.2347783)
Supplement: Lemke et al 2024 SOM_revised.docx [file KPSB_A_2347783_SM1698.docx]

*Table S1. Mutant lines used in study*

| Mutation / transgene | Gene Name / construct | Gene # | DNA/Protein Change | Back-ground ecotype | Notes | Ref. |
| --- | --- | --- | --- | --- | --- | --- |
| *acd2-2* | *ACCELERATED CELL DEATH 2 / RED CHLOROPHYLL CATABOLITE REDUCTASE* | *At4g37000* | G to A substitution in affecting splicing | Col-0 | Generated by ethyl methane-sulfonate treatment | ^1^ |
| *oxi1-1* | *OXIDATIVE SIGNAL-INDUCIBLE 1* | *At3g25250* | GABI_355H08 T-DNA in 2^nd^ exon | Col-0 |  | ^2^ |
| *pub4-6* | *PLANT U-BOX 4* | *At2g23140* | U-box domain amino acid substitution (G255R) | Col-0 | Generated by ethyl methane-sulfonate treatment | ^3^ |
| *pt-yb CD3-998* | *35S*::*TobaccoRBCS*(1-79)*-YFP* |  |  | Col-0 | Leads to plastid-localized YFP | ^4^ |

*Table S2. Primers used for PCR-based genotyping*

| Gene / T-DNA | Primer orientation / name | Sequence |
| --- | --- | --- |
| GABI-KAT Left Border | Gabi-KAT 03144 | ATATTGACCATCATACTCATTGC |
| *oxi1-1* (*Gabi_355H08*) | For. / JP1291 | CCTTTCCAAACAAAGCAAGTG |
|  | Rev. / JP1292 | AAGAAACGTCTCTTCCGCTTC |
| *pub4-6* dCAPS genotyping: *Hpa*II digestion, wt = 97 bp, mutant = 121 bp | For. / JP742 | TATTAGAGTAGTGTGAGTCAGG |
|  | Rev. / JP743 | GATCCAGTGATTGTGTCATCC |
| *acd2-2* dCAPS genotyping: *Pst*I digestion, wt = 124 bp, mutant = 149 bp | For. / JP1144 | GAGAATCTTAAAGTTTGTTTTGTTCTGCA |
|  | Rev. / JP1145 | TCGACCACAAAGTTTGGAGCT |

**References:**

1. Mach JM, Castillo AR, Hoogstraten R, Greenberg JT. The Arabidopsis-accelerated cell death gene *ACD2* encodes red chlorophyll catabolite reductase and suppresses the spread of disease symptoms. Proc Natl Acad Sci U S A 2001; 98:771-6.

2. Camehl I, Drzewiecki C, Vadassery J, Shahollari B, Sherameti I, Forzani C, et al. The OXI1 kinase pathway mediates Piriformospora indica-induced growth promotion in Arabidopsis. PLoS pathogens 2011; 7:e1002051.

3. Woodson JD, Joens MS, Sinson AB, Gilkerson J, Salome PA, Weigel D, et al. Ubiquitin facilitates a quality-control pathway that removes damaged chloroplasts. Science 2015; 350:450-4

4. Nelson BK, Cai X, Nebenführ A. A multicolored set of in vivo organelle markers for co-localization studies in Arabidopsis and other plants. Plant J 2007; 51:1126-36.
